# The impacts of low-carbon city pilot policies on natural population growth: empirical evidence from China’s prefecture-level cities

**DOI:** 10.3389/fpubh.2023.1214070

**Published:** 2023-07-14

**Authors:** Yaxin Zheng, Miao Zhang, Sen Wang, Lin Wang

**Affiliations:** ^1^College of Business and Economics, Australian National University, Canberra, ACT, Australia; ^2^China National Gold Group Gold Jewellery Co., Ltd., Beijing, China; ^3^School of Business, Operations and Strategy, University of Greenwich, London, United Kingdom; ^4^School of Management, Chongqing University of Technology, Chongqing, China; ^5^Institute of Digital and Intelligent Management, Chongqing University of Technology, Chongqing, China

**Keywords:** low-carbon city (LCC), low-carbon city pilot (LCCP) policy, natural population growth, carbon emissions, China

## Abstract

**Introduction:**

The carbon emissions that cities contribute drive the development of low-carbon cities (LCCs) and low-carbon city pilot (LCCP) policies. However, the lack of comprehensive understanding regarding the impacts of LCCP policies on natural population growth hampers effective policy design and implementation, thus constraining sustainable development at the city level.

**Methodology:**

Extending the existing papers which focus on the relations between low-carbon pilot policies and industry transformation or economic growth, this research applies several experimental methods [e.g., Propensity Score Matching-Difference in Differences (PSM-DID)] to investigate the impacts of low-carbon pilot policies on natural population growth by applying the data from 287 prefecture-level cities in China from 2003 to 2019.

**Results and Discussion:**

This research found that low-carbon pilot policies would positively influence the low-carbon cities’ natural population growth by influencing (a) economic factors, (b) political factors, (c) technological factors, and (d) the living environment. This research establishes a framework for understanding the impact mechanisms of LCCP on natural population growth. This paper investigates how industrial structure optimization, policy design and implementation in different regions, technological innovations, and urban green space theoretically affect natural population growth. This paper also proposed characteristics of LCCP which should be theoretically concerned by the government. From a practical perspective, this research suggests several policy recommendations. Central and local governments are encouraged to prioritize industrial structure optimization and assess populations’ dependence on cultivated land. Providing additional policy support to underdeveloped areas is crucial to promote the balance between economic and environmental development. Furthermore, establishing online public health platforms and urban green spaces is proposed to enhance the population’s health and complement the implementation of LCCP policies. This offers both theoretical and practical insights into the impacts of LCCP policies on natural population growth. Its findings contribute to designing and implementing LCCP policies in China and other developing countries at a similar development stage.

## 1. Introduction

In 2021, the urban population rate worldwide was 56% (1), highlighting the significance of rapid urbanization on global sustainable development (2). While contributing 70% of the global GDP, cities also produce 70% of global greenhouse gas emissions (3, 4) and have challenged the sustainable development of urban areas and the planet (5). Hence, cities are expected to develop economic, social and environmental conditions (6) to deal with the environmental and climate issues caused by urbanization (7), such as facilitating Low-carbon City (LCC) development (8) by introducing low-carbon city pilot (LCCP) policies (9). Developing low-carbon pilot cities is expected to achieve a win-win between economic growth and environmental protection (9). After all, economic growth should not be achieved by sacrificing the environment (10), which fits into the carbon reduction action plans made by countries worldwide (11).

The emergence of “Low-carbon Cities” (LCCs) is coined in response to the increasing demands for carbon emissions reductions and global warming alleviation (12–14). LCCs refer to a sustainable urbanization approach which connects the government, private sectors and civil societies to reduce cities’ carbon footprint by minimizing fossil fuel consumption (15). Since the concept of “low carbon” was first proposed in the UK energy white paper in 2003 (16), the low-carbon transformation in economic development and perceptions on consumption have been undertaken by cities to enhance competitiveness (6). For instance, approximately 60% of C40^1^ cities have set carbon reduction targets or developed action plans for climate change (13). To conclude, the policies for LCC development issued and implemented by governments worldwide (11) have highlighted the new directions for cities’ sustainable operations (8).

As the largest developing country (17) and carbon emitter of the world (18, 19), China has already set climate targets, such as achieving the “carbon peak” in 2030 and “carbon neutral” in 2060 (17). Based on Huang et al. (20), China’s low-carbon development aims to fulfill the transformation from “green low-carbon cities” to “high-quality cities,” which shift from low energy consumption/pollution to sustainable human settlements. Low-carbon City Pilot (LCCP) policy is always seen as a macro-level policy (21), which points out the direction for optimization of industrial and energy structure (22). Additionally, LCCP policy is argued to play a crucial role in combining environmental regulation tools at the micro level (23). Since 2017, over six provinces and 81 cities in China have been impacted by the LCCP policy (24), reflecting China cities’ initiatives to respond actively to climate change and low-carbon transformations (23).

The relationship between natural population growth and LCCP policies promoting economic and environmental development balance in China is still worth investigating because it determines whether the existing economic development pattern is sustainable (25). However, the existing LCCP-relevant studies mainly focus on policy analysis, design and evaluation to promote low-carbon economic and transport system development (20) rather than further investigate their impacts on natural population growth. After all, the LCCP policies marked by making cities’ economic and social development patterns more low-carbon-oriented (26) are not treating natural population growth as their central concern. Unfortunately, natural population growth is found critically influences economic growth [e.g., (27–29)] and the environment [e.g., (30)], although how it works is still under discussion (31). Additionally, based on scholars [e.g., (20)], it is still crucial for China to consider the pathways to optimize the policy mechanisms to facilitate the development of a greener and more liveable city, which can be seen as a new stage for eco-cities development in China. However, the existing studies [e.g., (32–34)] tend to focus on the impacts of LCCP policies on industry transformation and economic growth rather than investigating its impact mechanisms (35) from the LCC level (36). Consequently, the existing studies show limited insights into the relationships between LCCP policies and natural population growth, especially at the city level. Different from the previous studies investigating the impacts of LCCP policies from the industrial transformation or development perspective, this paper is expected to investigate the impacts of China’s LCCP policies on LCC’s natural population growth. Therefore, this paper is expected to theoretically contribute to (1) building and enriching a framework of LCCP policies’ impact mechanisms on natural population growth and (2) emphasizing the factors of LCCP policies the LCCs should consider. This research practically suggests several policy recommendations for policymakers to effectively implement the LCCP policies with a reasonable natural population growth, thereby contributing to LCCs’ sustainable development in China and offering successful examples for large carbon emitters worldwide to achieve city-level sustainable development.

This research makes efforts to use empirical methods to answer the question above. We collect data from 287 prefecture-level cities in China from 2003 to 2019. This paper uses LCCP policy as the core explanatory variable, and the natural population growth rate is used as the explanatory variable. Mechanism variables and control variables are also considered. Robustness tests, parallel trend tests, placebo tests, the Propensity Score Matching-Difference in Differences (PSM-DID), bacon decomposition, and modified DID estimation are used. Finally, heterogeneity and mechanism analyses are conducted to conclude that LCCP policies can promote natural population growth by influencing (a) economic factors, (b) political factors, (c) technological factors, and (d) the living environment. This research theoretically contributes to knowledge by building a framework of LCCP’s impact mechanisms on natural population growth based on the empirical findings above. Furthermore, this research theoretically enriches several crucial features of LCCP policies which should be considered to optimize the effects of LCCP on rational natural population growth, such as time-lag effects, LCC’s administrative level and locations, and supporting policy supplements. From the practical implication perspective, this research recommends that policymakers and enterprises smartly design and implement LCCP policies to contribute to rational natural population growth and achieve sustainable development.

This paper is structured as follows. In the literature review section, this paper starts with the concept of LCC and discusses the impacts of natural population growth on economic growth and the environment. Then, the impacts of LCCP policies on LCC’s natural population growth were discussed based on the factors driving natural population growth synthesized from extant studies. The data collection and analysis approaches are explained next. We then report the empirical findings of the statistical analysis, which clarifies the impacts of LCCP policies on natural population growth in the chosen sample LCCs. Last, the theoretical implications of this paper are discussed by comparing our findings with previous relevant studies and recommendations for future research are made after explaining the practical guidance brought by this research.

## 2. Literature review

**Figure 1:**
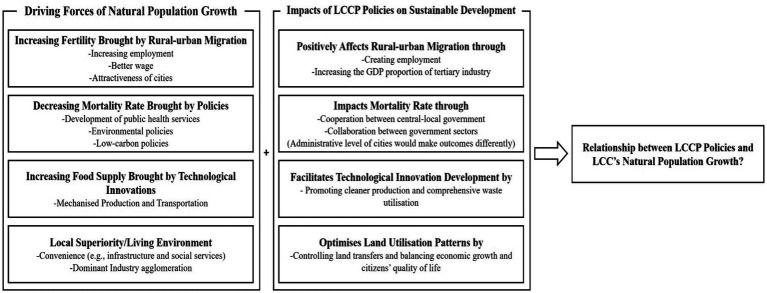
The logic of literature review.

This section can be divided into four parts. Section 2.1 focuses on the impacts of population growth on economic and environmental development to consolidate the necessity of this research. Section 2.2 synthesizes the driving forces of natural population growth from relevant papers, which was the basis for building links between LCCP policies and natural population growth in section 2.3. The logic of this section is to analyze the relationship between LCCP policies and natural population growth by discussing (1) what drives natural population growth and (2) how LCCP policies would affect the driving forces of natural population growth. After all, as limited studies are engaging in the impact mechanisms of the LCCP policies (35, 37) from the LCC-level perspectives (36), we can hardly build direct relationships between the above keywords. The figure below (see Figure 1) illustrates the logic of conducting the literature review:

### 2.1. Impacts of population growth on economic growth and environment

The relationship between population growth and economic development has been contentious in economics-relevant studies (31). Some scholars (28, 38–40) argue that population growth facilitates economic growth. For instance, Tiwari (40) and Aiyetan and Olomola (38) empirically found unidirectional causality among population growth, CO2 emissions and economic growth, which means that economic growth occurs accompanied by the increasing population and CO2 emissions. Similarly, Rahman et al. (28) reported that the population and economy grew simultaneously in China and the United States over the last 40 years. For instance, Zhang et al. (41) pointed out that the increasing urban population drives large-scale constructions of infrastructure and buildings in China, facilitating the development of the secondary industry. Furthermore, Bloom et al. (39) mentioned that population growth would positively affect economic growth through knowledge capital accumulation. Similarly, the neoclassical economics paradigm also regarded population growth as the catalyst of technological development and other positive changes (42). Furthermore, Park (43) found that population growth positively influences economic growth when the growth of population and per-capita GDP are independent.

Nevertheless, some papers (27, 29) found that negative population growth would benefit economic growth. For instance, unlike the opinions proposed by Bloom et al. (39) above, Bucci (27) argued that the shrinking population allows individuals to acquire new knowledge easier, enhancing economic growth by facilitating technological progress. Furthermore, population growth requires more cultivated land, driving more countries to issue cultivated land protection policies to support the growing population (44). However, Deng et al. (45) empirically found that the GDP developments of secondary and tertiary industries reduce cultivated land, which means that the population growth contradicts the developments of secondary and tertiary industries in some cases. Additionally, Sasaki and Hoshida (29) empirically found that the economy’s capital stock can be distributed to fewer individuals when the population decreases, increasing the per-capita income in the long term. Similarly, Gupta et al. (46) pointed out that the increasing population and child dependency ratios constrain countries’ investments in expanding production and reduce per-capita income.

On the other hand, some studies (27, 47) indicated that the correlation between population growth and economic growth is non-linear. Unlike Sasaki and Hoshida (29) and Gupta et al. (46), Christiaans (47) found that the correlation between population growth and economic growth is more complicated since the per-capita income grows only if the negative population growth rate can reach a particular level. Furthermore, the impacts of population growth on economic growth should also be determined by the degree of altruism of individuals toward future generations (27). Additionally, Lin et al. (48) empirically proved that although per-capita GDP positively influences per-capita carbon emissions, the correlation between population growth rate and per-capita GDP is insignificant.

The impacts of population growth on environmental development also link to sustainable development, which refers to “meeting the needs of contemporary people, but not endangering the development of future generations” (49). World population growth is a crucial factor in environmental degradation, making environmental change adaptation even more difficult (50). To specify, population growth threatens the arable land to produce food and settlement, bringing a mass of energy consumption and industrial wastes (42) and imposing substantial pressure on the environment (30). Wang and Yang (51) get a similar answer from a China-based empirical study as they argue that population growth increases the demand for goods and services and causes increasing pollutants and environmental deterioration (e.g., green areas’ decreases or damages). Therefore, decelerating population growth would contribute to sustained development by balancing economic growth and environmental protection. On the other hand, Kabisch and Haase (52) pointed out that offering citizens urban green space (UGS) would be a challenge since the increasing population influences housing development and urban planning.

Furthermore, to expand the UGS-relevant viewpoints above, existing scholarly papers have empirically demonstrated that population growth has an adverse impact on citizens’ physical and mental well-being by compromising the availability of UGS. For instance, Kondo et al. (53) revealed that UGS exposure negatively associates with mortality, heart rate and violence. Moreover, Nutsford et al. (54) emphasized that enhanced accessibility to UGS contributes to reducing treatment counts of anxiety/mood disorders, which means that the citizens would be more likely to experience mental issues when the UGS is converted into construction sites. However, the potential health risks of UGS were found by Wolch et al. (55) as they proposed that the UGS near the heavy traffic area may damage public health if interventions focus on encouraging walking and cycling without reducing air pollution.

Population growth contributes to climate change through excessive agricultural activities and fossil fuel consumption (56). For instance, the extreme coastal water levels caused by climate change severely impact the ecosystem of coastal zones and threaten people and infrastructures in these areas (57). Maja and Ayano (30) also pointed out that population growth increases deforestation and degradation, contributing to greenhouse gas emissions and exacerbating global warming. Moreover, human activities influence soil conditions, leading to extensive degradation and exhaustion (58). To sum up, demographic foresight (e.g., recognizing the future trajectories of the world population) matters for a more sustainable future (59).

However, Edeme and God (60) indicated that population reduction policies cannot always solve environmental issues since strong and quality institutions should be utilized as supplements, such as enforcing and implementing environmental laws. Additionally, the population always links to religious and cultural barriers, making population regulation a sensitive topic and difficult to implement (30).

After discussing the possible impacts of natural population growth on economic and environmental development, the next section will focus on the driving forces of natural population growth, which is the basis for building relationships with LCCP policies.

### 2.2. Driving forces of natural population growth

Natural population growth considers the average annual rates of births and deaths over a long term, which reflects a country’s/region’s population age structure (e.g., the aging population) (61, 62) and demographic dynamics (63). Since the natural population steadily evaluates the local demographic systems, it is always utilized to measure cities’ paths of socioeconomic transitions (64).

Natural population growth is responsible for urbanization, especially in the developing world (65). Based on relevant studies, natural population growth is caused by (1) the increasing fertility brought by the rural–urban migration (65–67), (2) the declining urban mortality rate (65, 68, 69), (3) the increasing urban food supply brought by technological innovations (68), (4) location superiority and decent residential environment (70). This section will outline and discuss the four aforementioned driving forces that influence natural population growth, as it is the foundation for examining the potential influence of LCCP policies on natural population growth via affecting these driving forces.

The rural–urban migration is the result of economic growth. Countries experienced rapid economic growth following World War II (68), which facilitated the growth of the urban labor market [e.g., the increasing urban employment (71) and wage (65, 72)] and made urban areas more attractive (73, 74). Consequently, the migration from rural areas would eventually change into urban-born populations and positively affect the fertility in urban areas, although urbanization cannot be seen as a single process (71). However, the natural population growth brought by migration would be slower because the labor market cannot digest fast-growing populations (67), which causes the re-distribution of population or even counter-urbanization (71). For example, Barreira et al. (66) found that the unemployment rate negatively affects population growth since households intend to find other cities.

Policies influence the decreasing urban mortality rate. Based on scholars (65, 68), the declining urban mortality rate is highly relevant to the development of public health service systems and epidemiological transition. Furthermore, to mitigate the negative effects of environmental pollution on human fertility (75), the environmental policies implemented by countries, such as air pollution prevention and control, reduce infant mortality and contribute to the natural population growth rate (76). Moreover, the low-carbon policies reduce chronic disease incidence and improve human health outcomes (77, 78)—reducing the local mortality rate. Unfortunately, the government’s ability to provide basic services would be constrained by rapid population growth, which would eventually reduce citizens’ life quality, such as suffering inadequate social resources and networks (67).

The increasing urban food supply is caused by technological innovations, such as mechanized production and the introduction of railways/road transportation (68), which further solve starvation and increase natural population growth. Garenne and Gakusi (79) noted that food riots and declining nutrition led to slower natural population growth in both rural and urban areas in the 1990s in Zambia. However, it is worth mentioning that the conflicts between food supply and urbanization should be further considered. After all, converting from arable land to urbanization land would enlarge the gap between food demand and supply (80) and threaten natural population growth. Moreover, as proposed by Ding (81), population growth would be greater than the food productivity of land and eventually fail to meet the food demand of human beings.

Location superiority/decent living environment is more likely a converging factor since Cai et al. (82) emphasize that the gaps in life quality and living environment between rural and urban areas drive rural people to move to cities, influencing population growth. The convenience and satisfying infrastructure attract potential residents and prevent the existing population from moving out—the location advantages brought by better social services (67), convenient transportation, and dominant industry agglomeration attract more migration (70) and contribute to natural population growth. Nevertheless, the city’s development and citizens’ quality of life should be balanced. Otherwise, the industries would occupy residential or arable land (70), negatively impacting natural population growth by constraining migration and food supply.

In this section, the four driving forces of natural population growth, namely (1) rural–urban migration, (2) decreasing urban mortality rate driven by policies, (3) more advanced technologies, and (4) location superiority, have been synthesized and discussed. In the next section, we will analyze how the LCCP policies would affect the aforementioned driving forces of natural population growth and further explore how LCCP policies would influence natural population growth.

### 2.3. Low-carbon city pilot policies and natural population growth

Low-carbon city pilot (LCCP) policy is a crucial low-carbon program of the world since it measures to what extent cities can achieve the goal of carbon prevention and control (83), especially in developing countries. LCCP policy intends to optimize governmental governance by diminishing the trend of sacrificing the environment for economic growth, such as supporting carbon reductions by fiscal expenditure and strengthening carbon emissions constraints on carbon emitters (10), and accumulating experiences for prompting low-carbon cities (LCCs) (11). Unfortunately, the impact mechanism of LCCP is not sufficiently revealed (35, 37). More importantly, when conducting carbon emission-relevant studies, the analysis at the LCCs level is limited (36). Hence, this section will synthesize the impacts of LCCP policies on sustainable development and then combines the findings in section 2.2 to further discuss the possible mechanisms of LCCP policies on natural population growth from the perspective of LCCs.

Zhang et al. (36) empirically found that LCCP policies would drive pilot cities to explore sustainable development patterns, such as developing modern financial industry and cultural and creative bases. Consequently, the pilot countries would achieve a win-win for economic development and environmental protection (9). Specifically, LCCP policies create employment (84) while increasing the GDP proportion of tertiary industry (36). Based on the findings in section 2.2, economic growth (e.g., better per-capita GDP and wages) would speed the rural–urban migration and, therefore, contribute to natural population growth.

However, some existing studies [e.g., (85)] also propose that the LCCP policies would influence the development of LCCs in different regions or at different development stages, thereby theoretically affecting the regional natural population growth differently. For instance, Wang et al. (86) discovered that cities in China with medium or better quality are predominantly concentrated in the Central and Eastern regions, which possess decent incomes and industrial structures, enabling more effective implementation of LCCP policies (87). As a result, the more appealing LCCs above would rapidly expand to saturation (88), thereby contributing to natural population growth. Comparatively, due to the limited fiscal capacity and limited policy leverage (89), the LCCs situated in the Western region are compelled to curtail their economic growth to comply with emissions reduction targets—which exacerbates the existing economic disparity with the Eastern regions (88). Consequently, the natural population growth would be negatively affected since labor forces exhibit reduced enthusiasm for migrating to the Western regions.

Combining the findings mentioned in section 2.2 and the earlier analysis, the different features and outcomes of LCCP policies implemented by LCCs would influence the natural population growth differently. LCCP policies emphasize central-local government cooperation and collaborations between different government sectors. For example, in responding to the central government’s action plans on low-carbon development, the local governments would introduce various policies and promote communications and collaborations between government sectors (35). More importantly, the local government at different administrative levels (89, 90) or development stages (91) would apply for different pilot programs, making the focus, goals, and enforcement degree different. As a result, the outcomes of economic growth and carbon emissions reductions would differ among LCCs with different administrative levels, influencing cities’ natural population growth differently. For instance, Yan et al. (91) found that the LCCP policies implemented in developed LCCs would more potentially reduce air pollution, driven by heightened awareness of personal health and environmental protection. Hence, LCCP policies are more likely to contribute to natural population growth by lowering the urban mortality rate in developed LCCs.

Interestingly, technological innovations brought by LCCP policies are not limited to agriculture (e.g., food supply) or transportation. Based on Yuan and Pan (10), LCCP policies require local enterprises to engage in technological innovations and stick to the sustainable operating paradigm. Consequently, local enterprises would promote cleaner production and comprehensive waste utilization (92) and restrain carbon emissions of particular LCCs. Based on the findings of section 2.2, the carbon emissions reductions brought by technological developments would and human health outcomes [Haines et al. (77, 92)] and contribute to natural population growth by reducing the local mortality rate. However, Yuan and Pan (10) also highlighted that the positive impacts of technological innovation brought by LCCP policies would only influence carbon emissions in the short term, which requires further investigations to discuss the relationships between LCCP policies and natural population growth.

LCCP policies can also achieve carbon emissions and optimize land utilization patterns by controlling land transfers (37). For instance, Lin et al. (48) empirically proved that the local governments of LCCs would withdraw the existing land of heavily polluting enterprises to optimize environmental conditions and facilitate sustainable development. As highlighted by section 2.2, the migration and food supply would be developed when the balance between economic growth and citizens’ quality of life is optimized (70), and hence, LCCP policies would contribute to natural population growth.

## 3. Model, variables, and data sources

### 3.1. Model

DID is a classic model for estimating the impact of external shocks (89), which can greatly reduce endogeneity issues. Noted Liu and Xu (94), based on the approval time of Lowcb (Note: in formulas, LCCP policy is presented as Lowcb), the cities implementing LCCP policies are the experimental group, and the remaining cities are the control group. Among them, the setting of the experimental group is based on the “Notice of the National Development and Reform Commission on Conducting Pilot Work in Low Carbon Provinces and Cities.” The DID model is set as follows:

(1) $$\mathrm{Birde}a_{\mathrm{it}}=\alpha_{0}+\alpha_{1}\mathrm{Lowc}b_{\mathrm{it}}+\alpha_{2}X_{\mathrm{it}}+\lambda_{t}+\mu_{i}+\varepsilon_{\mathrm{it}}$$

Birdea_it_ represents the natural population growth rate. Lowcb_it_ represents the dummy variable for the low-carbon city pilot. Among them, the experimental group is taken as 0 before the implementation of the policy, and 1 after the implementation, and all the control groups are taken as 0. X_it_ represents the control variables, λ_t_ and μ_i_ represent the year-fixed effect and city-fixed effect, respectively, and ε_it_ represents the random disturbance term.

This paper also performs a parallel trend test based on the coefficient of dynamic effect based on the DID model. The model is set up as follows: Lowcb^k^_it_ represents whether the sample of city i and year t is the kth year from the implementation of the policy.

(2) $$\mathrm{Birde}a_{\mathrm{it}}=\alpha_{0}+\overset{8}{\underset{k=-8}{\sum }}\alpha_{1,k}\mathrm{Lowc}b_{\mathrm{it}}^{k}+\alpha_{2}X_{\mathrm{it}}+\lambda_{t}+\mu_{i}+\varepsilon_{\mathrm{it}}$$

This paper studies the dynamic effect of each 8-year period before and after the policy, and the remaining variables have the same meaning as in the benchmark model. The confidence interval includes 0 when no significant difference exists between the experimental and control groups. The confidence interval does not include 0 when there is a significant difference between the experimental and control groups.

### 3.2. Variables

#### 3.2.1. Independent variable

In this paper, LCCP is used as the core independent variable. Effective environmental regulations positively affect people’s physical and mental health by improving the ecological environment, which in turn affects the natural growth rate of the population. Most studies [e.g., (76)] that have been conducted focus on the effects of environmental regulations or carbon emissions on mortality. However, limited studies have integrated measures of the effects on the natural population growth rate. As an important component of the population birth rate, improving the ecological environment also increases people’s fertility and intention.

#### 3.2.2. Dependent variable

The natural population growth rate is selected as the dependent variable in this paper, which is an important indicator of the rate of population growth and the development of population plans (57). The natural population growth rate equals the birth rate minus the mortality rate. The birth rate depends mainly on people’s fertility and willingness to have children. The mortality rate mainly depends on the economic status, ecological environment, medical conditions and other uncontrollable contingent factors. The mortality rate is widely applied to measure population growth in previous studies (95), while in this paper we consider both the birth rate and the mortality rate which aims to provide a more comprehensive view for measuring the population growth.

#### 3.2.3. Mechanism variable

This paper examines the transmission path of LCCP affecting the natural population growth rate from both macro and micro perspectives. In particular, green space is adopted as the mechanism variable for the measurement on the macro level. The impact of LCCP policies on citizens’ physical health (Health) and mental health (Confi) is assessed as the examination on the micro level. The objective is to determine whether LCCP policies mitigate the adverse effects of limited green area accessibility and subsequently influence natural population growth. The self-rated “healthy” or “very healthy” in the questionnaire is considered as physically healthy. On the contrary, it is physically unhealthy. The questionnaire with ‘little or no loss of confidence in oneself’ is considered as psychologically healthy. On the contrary, it is psychologically unhealthy.

#### 3.2.4. Control variable

In addition to the explanatory variables, other external factors may also affect the explained variables. If the effects of these potential factors are ignored, the regression results may be biased. Therefore, five control variables are selected in this paper: (1) Education level (Teastu), measured by the teacher-student ratio, and the higher the level of education, the lower people’s willingness to have children, which reduces the natural growth rate of the population (96). (2 and 3) Industrial structure (Gdp2p and Gdp3p) is measured by the share of secondary and tertiary industries to GDP. As mentioned in the literature review, the developments of secondary and tertiary industries contradict the natural population growth (45), which needs to be empirically tested; (4) economic development level (Gdpreaave), measured by real *per capita* GDP. In economically developed regions, people have higher material living standards and lower population mortality rates (97). It can also potentially examine whether the incomes would facilitate rural–urban migration (74), which is emphasized in the literature review; (5) the level of Internet development (Intpop), measured as Internet coverage. Chen and Liu (98) find that internet development significantly promotes people’s health, thereby promoting the natural growth rate of the population. To assess the impact of LCCP policies on natural population growth through the influence on the living environment, we introduced a variable green area (Green) apart from self-rated health (Health) and mental health (Confi) above. Micro control variables in the mechanism analysis section include gender, type of household, age, whether or not drinking alcohol, whether or not smoking, and whether or not having a job.

### 3.3. Data source

Considering the availability and accuracy of data, this paper is based on the data of 287 prefecture-level cities in China from 2003 to 2019 for research and analysis. The relevant data are mainly from the China Urban Statistical Yearbook, the China Urban and Rural Construction Statistical Yearbook and the China Labor-force Dynamics Survey (CLDS). For missing values, this paper uses interpolation to fill in the values by Stata. The descriptive statistics of the main variables involved are shown in Table 1.

**Table 1:** Descriptive statistics of variables.

Variable	Description	Unit	*N*	Mean	Std.Dev.	Min	Max
Birdea	Natural population growth rate	‰	4,879	5.626	4.743	−6.700	20.100
Lowcb	LCCP policies	--	4,879	0.173	0.378	0.000	1.000
Teastu	Teacher-student ratio	Ratio	4,879	0.072	0.017	0.041	0.125
Gdp2p	Secondary industry to GDP	%	4,879	47.441	10.949	19.760	77.220
Gdp3p	Tertiary industry to GDP	%	4,879	38.585	9.358	17.330	68.560
Gdpreaave	Real *per capita* GDP	Deflated	4,879	1.993	2.243	0.058	11.607
Intpop	Internet penetration rate	Household/100 people	4,879	14.489	14.475	0.543	76.685
Green	Garden green area	km^2^	4,879	60.073	121.068	1.780	916.740
Health	Self-rated health	--	16,767	0.608	0.488	0.000	1.000
Confi	Mental health	--	16,767	0.690	0.462	0.000	1.000

## 4. Empirical results

### 4.1. Impact of LCCP policies on natural population growth

This paper uses the DID method to study the impacts of LCCP on the natural population growth rate, and the results are shown in Table 2. The coefficient of LCCP is positive when city and year fixed effects are included in column (1)—control variables are not included. It indicates that the natural population growth rate in low-carbon pilot cities increases after the policy is implemented compared to non-pilot cities. In other words, implementing the LCCP helps improve the natural population growth. There is no significant change in the promoting effect of LCCP on the natural population growth rate when the control variables of social level and economic level are, respectively, added in columns (2) and (3). Compared to the previous empirical findings, the coefficient of LCCP decreases after the inclusion of control variables, indicating that the social and economic level control variables affect the natural population growth rate to some extent. The coefficient of the core explanatory variable increases when all control variables (except fixed effects) are added in column (4). The coefficient of LCCP is significantly positive at the 1% level when all control variables and fixed effects are included in column (5). It can be seen that the higher the level of education, the lower the natural population growth rate. The higher the Internet coverage, the level of industrial structure and the real *per capita* GDP, the higher the natural population growth rate. Additionally, the inclusion of control variables raises the adjusted R^2^, indicating that the selected control variables are effective.

**Table 2:** DID regression results.

	(1)	(2)	(3)	(4)	(5)
Lowcb	0.7998^***^ (0.1887)	0.7023^***^ (0.1899)	0.7679^***^ (0.1891)	1.0282^***^ (0.2268)	0.7167^***^ (0.1893)
Teastu		−17.9385^**^ (8.4244)		−96.0471^***^ (5.6673)	−15.5957^*^ (8.7038)
Tntpop		0.0595^***^ (0.0090)		0.0213^***^ (0.0082)	0.0508^***^ (0.0103)
Gdp2p			0.0899^***^ (0.0184)	0.0011 (0.0086)	0.0981^***^ (0.0186)
Gdp3p			0.0973^***^ (0.0295)	−0.0138 (0.0130)	0.0971^***^ (0.0296)
Gdpreaave			0.3084^***^ (0.0368)	0.1832^***^ (0.0351)	0.2242^***^ (0.0398)
Constant	5.4974^***^ (0.0576)	5.9384^***^ (0.5816)	−3.1506 (1.9172)	12.1800^***^ (0.8602)	−2.9638 (2.2409)
Year FE	Yes	Yes	Yes	No	Yes
City FE	Yes	Yes	Yes	No	Yes
N	4,879	4,879	4,879	4,879	4,879
Adjusted R^2^	0.5653	0.5714	0.5721	0.0867	0.5761

Robust standard errors are corrected in parentheses. ***, **, and * represent statistical significance at the 1%, 5%, and 10% levels, respectively.

### 4.2. Robustness test

#### 4.2.1. Replacement of model and sample

The following tests are conducted in this paper to ensure sound robustness of the results. Since policies may have time lags, this paper first lags LCCP by one and two periods, respectively, and then performs DID regressions. According to the results in columns (1) and (2) of Table 3, the promoting effect of LCCP on the natural population growth rate increases as the number of lags increases. It indicates that LCCP has a greater promoting effect on natural population growth over time.

**Table 3:** Results of model and sample replacement.

	(1)	(2)	(3)	(4)	(5)	(6)
L. Lowcb	0.5828^***^ (0.2021)					
L2. Lowcb		0.8444^***^ (0.2139)				
Lowcb			0.7167^**^ (0.3076)	0.7167^*^ (0.3719)	0.5875^***^ (0.2100)	
Cbtrad						3.2745^***^ (0.3228)
Constant	−3.2880 (2.4182)	−3.8538 (2.4687)	−2.9638 (4.0820)	−2.9638 (3.1993)	−6.3193^***^ (1.7609)	−1.8015 (2.2063)
Control variables	Yes	Yes	Yes	Yes	Yes	Yes
Year FE	Yes	Yes	Yes	Yes	Yes	Yes
City FE	Yes	Yes	Yes	Yes	Yes	Yes
N	4,592	4,305	4,879	4,879	4,352	4,879
Adjusted R^2^	0.5836	0.5918	0.5760	0.5760	0.5895	0.5868

Robust standard errors are corrected in parentheses. ***, **, and * represent statistical significance at the 1%, 5%, and 10% levels, respectively.

Second, cities and years were clustered separately in the regression. When clustering cities, it is assumed that the years of the same city are disturbed by common factors, and the random disturbances between different cities are not correlated. When clustering the years, it is assumed that the cities of the same year are disturbed by common factors, and the random disturbances between different years are not correlated. From the results in columns (3) and (4), it is clear that LCCP still significantly affects the natural population growth rate. When adding clustering standard errors, the assumed conditions are stricter, resulting in a decrease in significance.

Next, municipalities directly under the central government and provincial capitals are excluded. Compared with other prefecture-level cities, cities with higher administrative levels have different carbon emission control systems, fertility subsidy policies, and health care coverage. To eliminate the interference of this specificity, this paper regresses using the sample without municipalities directly under the central government and provincial capitals. The results in column (5) show that these cities with special administrative levels basically do not change the positive correlation between LCCP and natural population growth rate. Due to the higher health level of cities with higher administrative levels, the marginal utility of LCCP policies is smaller.

Finally, the core explanatory variable is replaced. Since carbon emission trading pilot and PLLC have similar policy impacts, this paper also replaced the DID variable to consider eight provinces and cities (e.g., Beijing, Tianjin, Shanghai, Chongqing, Shenzhen, Guangdong, Hubei, and Fujian) as the experimental group. The results are shown in column (6). It can be seen that the carbon emissions trading pilot is significantly and positively related to the natural population growth rate. It is because the carbon emissions trading pilot improves carbon efficiency and, to some extent, promotes low-carbon goals’ achievements.

#### 4.2.2. Parallel trend test

**Figure 2:**
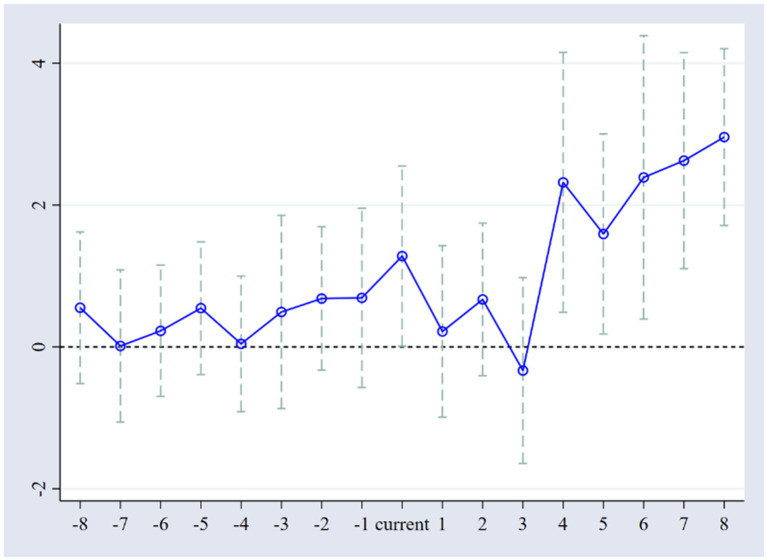
Parallel trend test results.

If there is a certain difference between the processing group and the control group beforehand, then using DID results can no longer represent the net effect of the policy. The assumption of parallel trends is a prerequisite for conducting DID analysis. In order to visually test the effect of LCCP on the natural population growth rate, the dynamic effect coefficient of this policy shock is estimated in this paper, and the results are shown in Figure 2. It can be seen that before the implementation of LCCP, there was no significant difference between the experimental and control groups from the perspective of the natural population growth rate. The confidence interval does not contain 0 within 4 years after the policy implementation. The empirical findings above indicate that the data used in this paper satisfy the parallel trend assumption and that there is a 4-year lag in the effect of LCCP on the natural population growth rate, further demonstrating the robustness of the previous results. It may be due to the time required to implement LCCP and the time lag for the full effect of the policy.

#### 4.2.3. Placebo test

**Figure 3:**
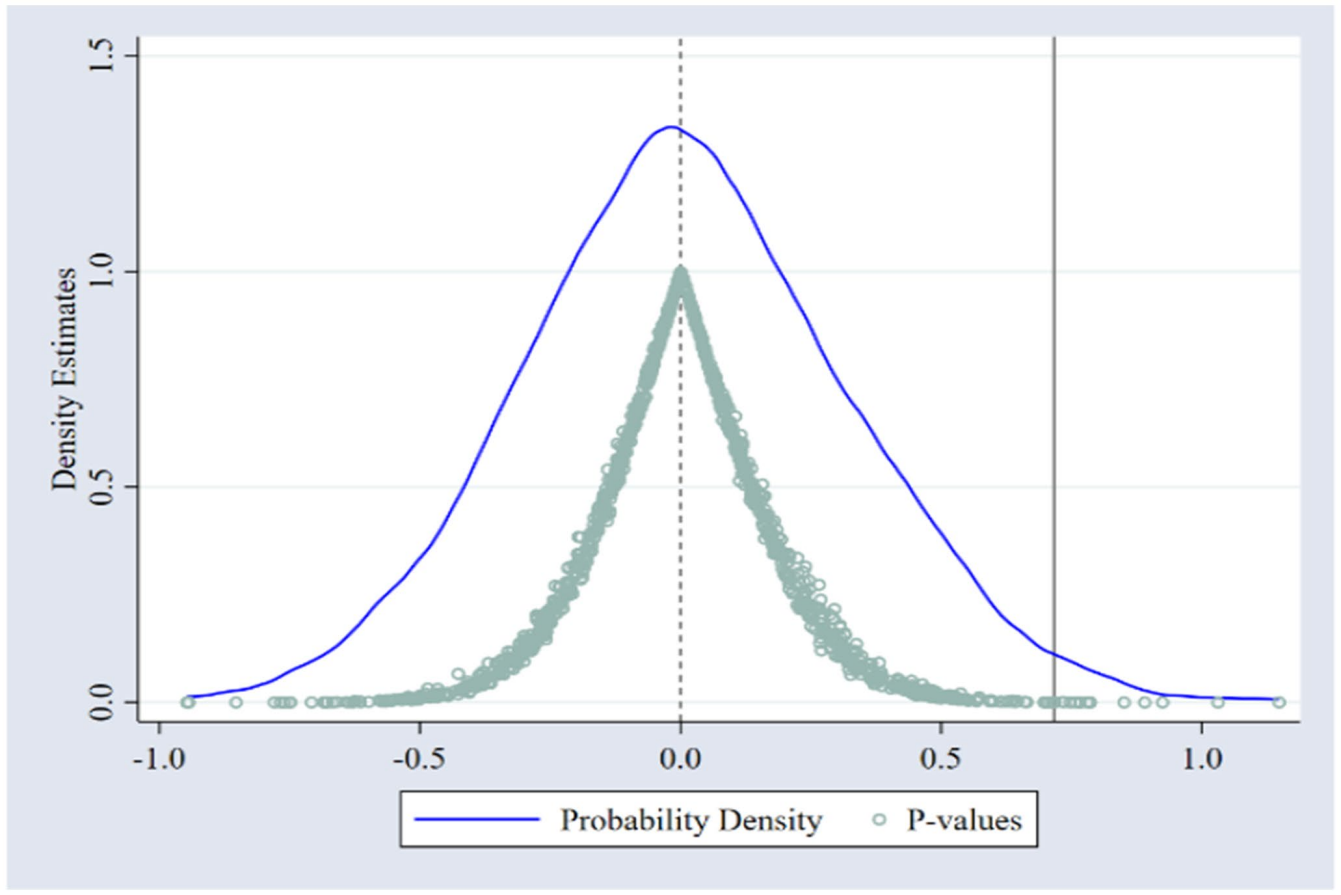
Placebo test results.

To further test the validity of LCCP, this paper uses the placebo test as follows. First, this paper randomly sorts the experimental group and policy time 1,000 times simultaneously to obtain 1,000 simulated policy variables. The simulated variables are sequentially put into the original regression model to test whether the mean of these effects is equal to 0 to determine whether the results of the benchmark regression are obtained by chance. The coefficients and *p*-values of the simulated policy effects are shown in Figure 3. As can be seen, the true effect of the benchmark regression is located at the end of the right tail, which indicates that the results of the benchmark regression are not found by chance.

#### 4.2.4. PSM-DID

**Figure 4:**
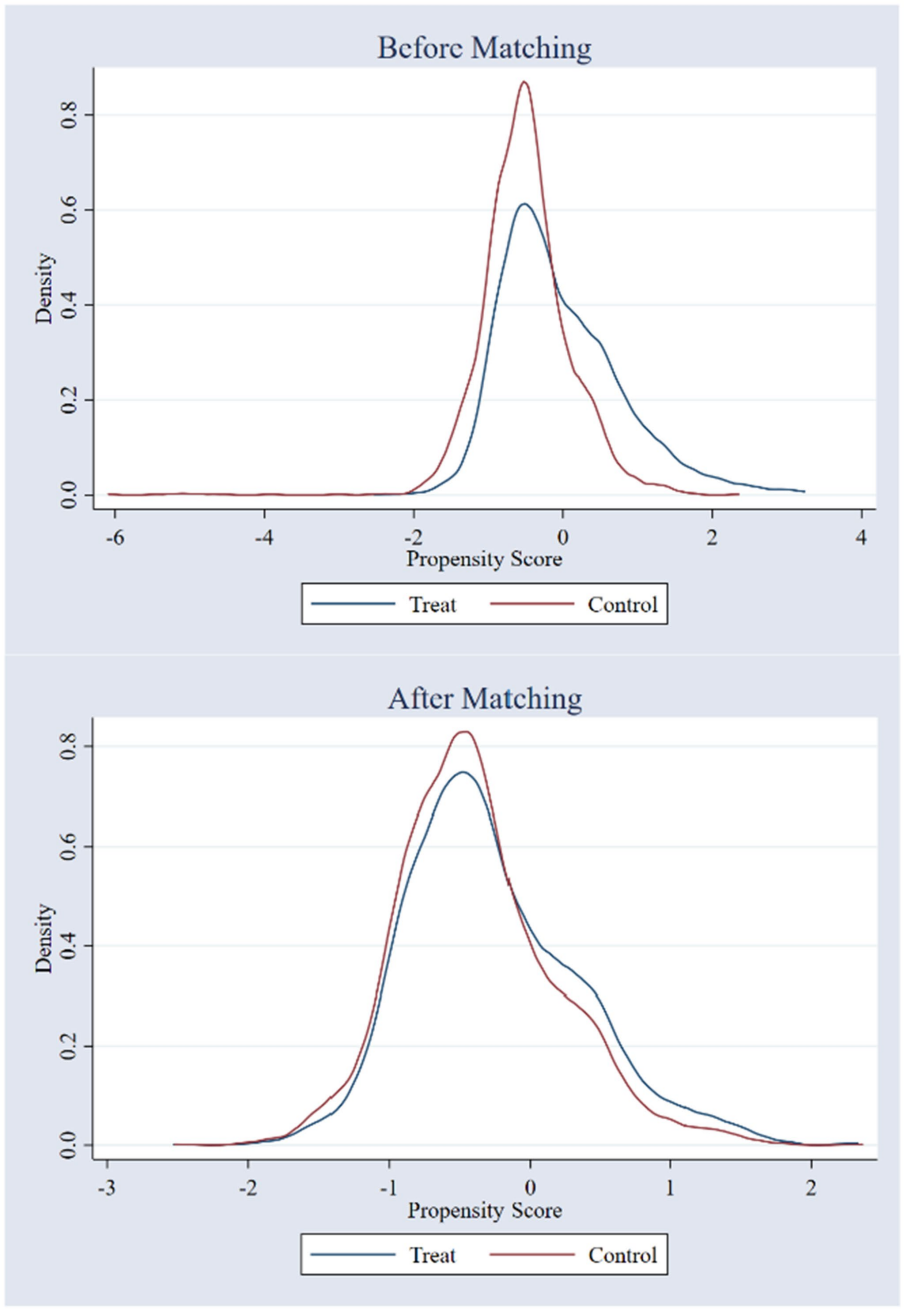
Nuclear density distribution before and after matching.

Since LCCP is based on local declarations and the representativeness of pilot layouts, the policy is not completely randomized but is closely related to factors such as the economic development status of the city. This paper uses PSM to obtain a control group corresponding to the experimental group to avoid the bias of DID estimation results. The matched results are shown in Table 4, and there is no significant difference in the covariate characteristics between the experimental and control groups. As expected, the unmatched results show significant differences between the experimental and control groups in terms of education level, industrial structure, real *per capita* GDP, and Internet coverage. According to Figure 4, the kernel density functions before and after matching are much closer, largely reducing the interference caused by selection bias.

**Table 4:** Balance test results of matching characteristic variables.

Variable	Unmatched matched	Mean		%Bias	*t*-test	
		Treated	Control		t	*p* > |t|
Teastu	U	0.074	0.071	14.8	5.14	0.000
	M	0.073	0.074	−2.3	−0.75	0.452
Gdp2p	U	47.124	47.729	−5.5	−1.87	0.061
	M	47.439	47.318	1.1	0.36	0.720
Gdp3p	U	41.113	36.711	46.5	16.24	0.000
	M	40.421	40.162	2.7	0.84	0.400
Gdpreaave	U	2.236	1.887	13.8	4.79	0.000
	M	2.153	2.146	0.2	0.09	0.931
Intpop	U	18.452	11.728	44.3	15.83	0.000
	M	16.561	17.282	−4.8	−1.45	0.146

After excluding selectivity bias, this paper performs regressions using samples with no null weights, respectively meeting the common support assumption and frequency weighting. The results are shown in columns (1)–(3) of Table 5. Compared with the benchmark regression, the coefficient of LCCP is smaller in the results of PSM-DID, indicating that the benchmark regression overestimates the effect of LCCP on the natural population growth rate, but this does not affect the robustness of the conclusions.

**Table 5:** PSM-DID regression results.

	(1)	(2)	(3)
Lowcb	0.3797^*^ (0.2235)	0.5731^***^ (0.1927)	0.4591^**^ (0.1991)
Constant	−7.0882^***^ (2.1404)	−3.7031^*^ (2.1228)	−6.5732^***^ (1.7014)
Control variables	Yes	Yes	Yes
Year FE	Yes	Yes	Yes
City FE	Yes	Yes	Yes
*N*	3,404	4,787	5,617
Adjusted R^2^	0.5864	0.5777	0.6151

Robust standard errors are corrected in parentheses. ***, **, and * represent statistical significance at the 1%, 5%, and 10% levels, respectively.

#### 4.2.5. Bacon decomposition

The problem of bias in staggered DID with Two-Way Fixed Effects (TWFE) has been discussed in the literature (99). Since the treatment effects of TWFE regressions are typically heterogeneous across experimental groups or policy times, the problem of using bad treatment groups and the appearance of negative weights may arise. Therefore, this paper refers to the Goodman-Bacon (100) decomposition of the DID estimator to examine the degree of bias in the staggered DID estimates under TWFE. The results are shown in Table 6. The first round decomposition included all control variables, and the anticipated good treatment effect was 1.0809 with a weight of 0.8057. The coefficient of LCCP remains significant in the second round of detailed decomposition without any control variables, and the anticipated good treatment effect was 1.0589 with a weight of 0.8477. Since the estimates of the bad treatment effect are all negative and the weights are small, the core findings of this paper can be considered robust.

**Table 6:** Bacon decomposition results.

First. considering control variables	Beta	Weight	Second. Without control variables	Beta	Weight
Estimate	0.7166^**^ (0.3076)	1.0000	Estimate	0.8130^**^ (0.3427)	1.0000
Treated	−0.5765	0.1609	Earlier vs. Later	−0.6012	0.1058
Within	−1.8424	0.0333	Later vs. Earlier	−0.4503	0.0465
Treated vs. never treated	1.0809	0.8057	Treated vs. never treated	1.0589	0.8477

Standard errors are in parentheses. ***, **, and * represent statistical significance at the 1%, 5%, and 10% levels, respectively.

#### 4.2.6. Modified DID estimation

This paper uses the two-stage estimation framework to identify, removing group and period effects in the first stage and obtain the average treatment effect in the second stage (101). The results are robust when the treatment effects are staggered and heterogeneous. According to the regression results in Table 7, LCCP still has a significant promoting effect on the natural population growth rate, indicating the robustness of the results from the benchmark regression.

**Table 7:** Two-stage estimation results.

Coefficient	Std. Err.	Z	*P* > |z|	95% conf. interval
0.9878	0.3298	2.99	0.003	[0.3413, 1.6342]

### 4.3. Robustness test

The results suggest that China’s LCCP policies significantly promote natural population growth. However, do the effects of population growth under the influence of other policies still exist? Are there significant differences between different environments and regions? To this end, the following heterogeneity analysis is conducted in this paper.

#### 4.3.1. Heterogeneity of healthy city pilot

In this paper, firstly, according to the “Notice of the National Office of Health Care on the Piloting of Healthy Cities” issued by the National Administration of Disease Prevention and Control in 2016, the cities implementing the healthy pilot are used as the experimental group, and other cities are used as the control group. The dummy variable of the healthy city pilot interacted with LCCP and then regressed. The results in column (1) of Table 8 showed that the variable Lowcb * Healcity have a facilitative effect at the 1% significance level, indicating that in those cities that implemented both LCCP and the healthy pilot, the two policy effects are not conflicting or contradictory, but mutually reinforcing. The Healthy City Pilot has a synergistic effect with LCCP policies to some extent by continuously improving the natural environment, social environment, and health services.

**Table 8:** Heterogeneity analysis results.

	(1)	(2)	(3)	(4)	(5)
Lowcb	0.6376^***^ (0.1929)	0.3958^*^ (0.2124)	−2.3736^***^ (0.3754)	0.5534^***^ (0.2086)	0.5335^***^ (0.2039)
Lowcb * Healcity	1.2276^***^ (0.4749)				
Lowcb * Healinf		1.3826^***^ (0.3468)			
Lowcb * East			4.3182^***^ (0.4481)		
Lowcb * Mid			3.4104^***^ (0.4920)		
Lowcb * West			2.6294^***^ (0.4608)		
Lowcb * Admi				0.9225^**^ (0.3738)	
Lowcb * Tier					1.3136^***^ (0.3905)
Constant	−3.1377 (2.2267)	−2.7503 (2.2430)	−1.0719 (2.2425)	−3.0450 (2.2302)	−2.9627 (2.2400)
Control variables	Yes	Yes	Yes	Yes	Yes
Year FE	Yes	Yes	Yes	Yes	Yes
City FE	Yes	Yes	Yes	Yes	Yes
N	4,879	4,879	4,879	4,879	4,879
Adjusted R^2^	0.5765	0.5772	0.5827	0.5764	0.5767

Robust standard errors are corrected in parentheses. ***, **, and * represent statistical significance at the 1%, 5%, and 10% levels, respectively.

#### 4.3.2. Heterogeneity of health informatization

Then, according to the national health informatization development index in 2022, this paper sets the dummy variable corresponding to the Top 60 cities to 1 and the other cities to 0. The dummy variable interacted with LCCP and then regressed; the results are shown in column (2) of Table 8. In cities with high levels of health informatization, LCCP can better promote population growth through good health system construction and application.

#### 4.3.3. Regional heterogeneity

To further investigate the regional heterogeneity of LCCP policies, this paper divides the sample into four groups: Eastern, Central, Western, and Northeastern regions. The generated dummy variables interacted with LCCP, respectively, before regression. Among them, the Northeast region is used as the control group. The results in column (3) of Table 8 showed that LCCP policies have the largest promoting effect on the natural population growth rate in the East, followed by the Central and Western regions. It may be because the scale of carbon emissions in the industrial development process is larger in the Eastern region. Hence, LCCP policies had a stronger emission reduction effect in this region and a stronger promoting effect on the natural population growth rate. In contrast, there are fewer industrial enterprises in the Central and Western regions, so the effect of LCCP on the natural population growth rate is limited.

#### 4.3.4. Heterogeneity of administrative levels

Cities at different administrative levels have different policy implementation plans. In general, cities with higher administrative levels have more resources, and the intensity of their policy enforcement may be higher. Therefore, the dummy variable interacted with LCCP and then regressed. Among them, the dummy variable corresponding to municipalities directly under the central government and provincial capitals is set to 1, and 0 for other cities. The results in column (4) of Table 8 indicate that the promoting effect of LCCP on the natural population growth rate is greater in cities with higher administrative levels, confirming the previous hypothesis.

#### 4.3.5. Heterogeneity of development level

Finally, this paper generated interactions between LCCP policies and the dummy variable measuring the level of urban development based on the New Tier 1 Cities Institute’s “2021 City Business Attractiveness Ranking” and a regression analysis was conducted. The corresponding dummy variable was set to 1 for Tier 1 and New Tier 1 cities and 0 for others. The results in column (5) of Table 8 showed that the promoting effect of LCCP on the natural population growth rate is greater in cities with higher development levels. In recent years, the natural population growth rate has been low due to the high cost of childbirth in Tier 1 cities. And implementing LCCP policies can reduce the gap in population growth between different regions.

## 5. Mechanism analysis

The results of this paper show that when cities implement LCCP policies, the natural growth rate of the local population increases, and how it works. Next, this paper examines macro and micro perspectives, specifically, the greening rate and physical and mental health. The results in Table 9 show that LCCP policies expand the urban green area. Increasing green space reduces the probability of stress-related problems, depression, and other mental illnesses. In addition, Richardson and Mitchell (102) find that an increase in green space areas reduces mortality rates from cardiovascular and respiratory diseases. On the other hand, LCCP policies improve people’s mental health and reduce the tendency of unnatural death, such as suicide. Research has shown that depression is an important factor affecting fertility and the number of children (103). People gain a sense of well-being while also increasing their willingness to have children. It has been shown that reducing carbon emissions has saved many people from early death or enabled people to live longer. The improvement of physical health through the LCCP policies has also improved fertility.

**Table 9:** Mechanism analysis results.

	Green area	Health	Confidence
Lowcb	19.1227^***^ (2.6996)	1.2280^***^ (0.3814)	0.9459^***^ (0.3662)
Constant	66.1088^***^ (14.4246)	2.0476^***^ (0.3482)	0.3362^***^ (0.3934)
Control variables	Yes	Yes	Yes
Year FE	Yes	No	No
City FE	Yes	No	No
City#Year FE	No	Yes	Yes
County FE	No	Yes	Yes
N	4,879	16,767	16,767
R^2^	0.9081	0.1519	0.0970

Robust standard errors are corrected in parentheses. ***, **, and * represent statistical significance at the 1%, 5%, and 10% levels, respectively.

## 6. Discussion and conclusion

Based on the existing studies [e.g., (14, 83)], developing and implementing LCCP policies are the keys to the low-carbon economy and natural population growth plays a crucial role in economic growth and the environment (28, 56). Hence, this research further investigates the impacts of LCCP policies on natural population growth since there is still a lack of research exploring the impact mechanisms of LCCP policies (37) from the LCCs’ perspectives (36).

### 6.1. Theoretical implications

This research contributes to knowledge by (1) building and enriching a framework of LCCP policies’ impact mechanisms and (2) theoretically emphasizing the features of LCCP policies from the perspective of LCCs’ natural population growth.

First, this research empirically found that the LCCP policies would influence LCC’s natural population growth by impacting (a) economic factors, (b) political factors, (c) technological factors, and (d) the living environment, which builds and enriches the under-investigated impact mechanism of LCCP policies.

- This research aligns with previous studies [e.g., (36)] by supporting that the LCCP policies promote natural population growth by optimizing LCCs’ industrial structure and the subsequent increase in per-capita income. This finding highlights the theoretical significance of optimizing industrial structure in establishing and developing LCCs. After all, the optimization of the industrial structure, as indicated by the rising contribution of the secondary and tertiary industries to the GDP, fosters employment opportunities in both secondary and tertiary industries (104), thereby accelerating rural–urban migrations by making the LCCs more attractive (73, 74). However, this research does not empirically establish the negative correlation between the development of secondary and tertiary industries and population growth, as argued by Deng et al. (45) and Wu et al. (44). A possible explanation noted by Shi et al. (105) suggests that scientific agricultural cultivation promotes effective land utilization, which would reduce dependence on cultivated land by the population.
- From the political perspective, this research highlights the varied impacts of LCCP policies on regional population growth, considering factors such as the administrative levels (90) and geographical locations (85) of LCCs. Significantly, this research extends the above research directions by further explaining the underlying mechanisms through how LCCP policies influence population growth, with a focus on regional differences. Consistent with previous studies [e.g., (87, 89)], this research suggests that the LCCP policies affect China’s Eastern and Western regions differently. The intensity of LCCP policies in Eastern China would be higher to align with the large carbon emissions basis and a greater willingness for industrial structure transformation (34, 88). Conversely, the LCCs in Western China are more likely to compromise with economic growth due to limited funding and policy leverage, as Fu et al. (89) noted. Consequently, this research empirically provides that the LCCP policies have more pronounced effects on improving air conditions in developed LCCs in Eastern China than in Western regions. Hence, the LCCP policies in Eastern China are more likely to contribute to population growth by reducing mortality rates.
- This research enhances the framework of the impact mechanisms of LCCP policies on natural population growth by proposing additional technological factors. By extending the technological factors driving population growth mentioned by existing papers which focus on food supply (68), transport system development (20) and sustainable operating paradigm (10, 33), this paper empirically found that the Internet penetration rate also positively impacts LCCs’ natural population growth. A possible explanation would be given by Bessière et al. (106), who mentioned that the Internet’s growth makes obtaining medical information and building interpersonal communications easier. Consequently, the informal support offered by the Internet (107) would reduce mortality by promoting better public healthcare and mitigating the negative effects of depression.
- This research provides a novel empirical approach proving the positive impacts of an increasing greening rate brought by LCCP policies on natural population growth. Unlike previous studies [e.g., (25, 108)] based on the ratio of urban green areas to the population, this research examines the self-rated physical and mental health of the citizens residing in Chinese prefecture-level cities. According to our empirical investigation, this research demonstrates that the increased green areas brought by LCCP policies alleviate individuals’ pressures, reduce the likelihood of depression, and consequently enhance overall health conditions and well-being. This finding further consolidates the existing viewpoints from studies [e.g., (91, 108)], underscoring that LCCP policies enhance urban green areas, leading to an improved living environment and reduced carbon emissions.

Second, to further serve the impact mechanisms of LCCP policies, this research theoretically proposes features of LCCP policies which should be cautiously considered during implementation on the LCC basis. This research empirically found a 4-year lag in the effects of LCCP policies on LCC’s natural population growth, which should be seen as a challenge in implementing policies (109). Moreover, as mentioned earlier, regional differences should be cautiously considered as this paper empirically found different carbon reduction outcomes when the LCCP policies are implemented in China’s LCCs in different regions. Additionally, this research empirically found that the LCCP policies can be combined with other supporting public policies [e.g., emission trading (110) and healthy city pilot (111)] to further facilitate LCCs’ natural population growth.

### 6.2. Practical guidance

This research makes substantial practical contributions to sustainable development and the promotion of rational population growth by further explaining the insights into the impact mechanisms of LCCP policies for China and other developing countries in similar development stages. First, this research recommends that both central and local governments in China prioritize the process of industrial structure optimization. LCCs are expected to develop cities’ attractiveness by increasing the proportion of the tertiary industry and employment level. For instance, it is suggested to promote the GDP contributions of service-relevant industries and encourage rational rural–urban migrations to make the LCCs more attractive. This approach applies not only to the more developed Eastern but also to the Western regions of China. Furthermore, the recommendation above also holds value for other developing countries that share similar development stages with China.

Second, this research helps policymakers in China and other developing countries to better understand that the formulation of LCCP policies should follow LCCs’ regional characteristics and administrative levels to make the goals and processes appropriate. For instance, this research recommends that the central government allocates increased policy support to the LCCs in underdeveloped areas (e.g., Western China). This support can take the form of financial investment in low-carbon infrastructures and the provision of tax breaks, aiming to alleviate concerns among underdeveloped areas regarding the sole pursuit of economic development. Moreover, this research suggests that the LCCs’ governments to further develop LCCP policies’ supporting policies to optimize their effects on sustainable development and public health services, such as developing emissions trading systems and healthy city pilot policies. Meanwhile, the LCCs’ governments and other developing countries should recognize and mitigate the time lag effects of LCCP policies. For instance, the LCCP policy design should be flexible to accommodate potential changes during implementation. Additionally, establishing a robust real-time monitoring evaluation system is crucial to identify the time-lag effects and to facilitate timely corrective actions.

Third, this research proposes that central and local governments, along with the enterprises in China, further develop low-carbon-relevant technological innovations when designing LCCP policies. This recommendation enables an in-depth promotion of sustainable development and rational population growth. Suggested technological innovations include optimizing transportation infrastructure, fostering cleaner production approaches, and undertaking comprehensive waste utilization. Moreover, facilitating technological innovations in agriculture is essential to increase the utilization effectiveness of cultivated land to better meet the population’s needs. Significantly, to highlight the importance of more effectively utilizing the Internet, this paper suggests that China’s central and local governments proactively establish official or authorized online healthcare communities or platforms, such as smartphone applications based on big data. These platforms can provide citizens with a wealth of medical information and improve the accessibility to quality public healthcare services, thereby complementing the implementation of LCCP policies.

Fourth, this paper recommends that the governments of LCCs in China and other developing countries enhance citizens’ physical/mental well-being and living environment by expanding the urban green spaces. However, it is crucial that the selection of green space locations aligns with scientific city planning principles. For instance, it is suggested to avoid establishing green spaces in close proximity to heavily trafficked areas. Furthermore, supporting policies promoting air pollution prevention and fostering a green lifestyle should be advocated to reinforce the positive impact of green spaces. By enhancing citizens’ physical/mental well-being, the LCCs in China and other developing countries can unlock the potential of LCCP policies and foster rational natural population growth.

### 6.3. Limitations and recommendations

Despite crucial insights proposed by this research, this research has limitations which should be further explored in future studies. First, as a China-focused study, it cannot always reflect the features of LCCs and LCCP policies of other countries in the developing or developed world. Hence, further studies are required to support other developing countries’ sustainable development and rational population growth. Second, although this research investigated the impacts of LCCP policies on LCCs’ natural population growth, the discussion on how LCCP policies should be implemented to achieve a sound/appropriate natural population growth rate should be further conducted in the future. For instance, this research recommends that optimizing industrial structure is crucial. However, this research also highlights the need to further explore the relationship between the development of secondary/tertiary industries and population growth, considering the population’s dependence on cultivated land. Furthermore, this research acknowledges the time-lag effects in policies, which the existing studies have substantiated. Consequently, further studies are expected to concentrate on mitigating the time-lag effects, ensuring timely responses to potential changes or challenges that may arise while implementing LCCP policies. The potential research directions above will contribute to the effective design and implementation of LCCP policies. Third, since the framework of the impact mechanisms of LCCPs is newly built and limited studies have touched on this aspect previously, the richness and comprehensiveness of the framework can be further investigated in the future, which reveals other future research directions. For instance, future research can measure other influential factors which impact the relationship between LCCP policies and natural population growth from the perspective of LCCs.

## Data availability statement

The original contributions presented in the study are included in the article/supplementary material, further inquiries can be directed to the corresponding author.

## Author contributions

YZ and MZ provided the research ideas, conceived the research model, and were responsible for data collection and analysis. SW produced the introduction, literature review, discussions, and conclusion and edited the manuscript. LW was responsible for research idea and critically revised the manuscript. All authors contributed to the article and approved the submitted version.

## Funding

This work was funded by the National Natural Science Foundation of China (72074037).

## Conflict of interest

MZ was employed by China National Gold Group Gold Jewellery Co., Ltd.

The remaining authors declare that the research was conducted in the absence of any commercial or financial relationships that could be construed as a potential conflict of interest.

## Publisher’s note

All claims expressed in this article are solely those of the authors and do not necessarily represent those of their affiliated organizations, or those of the publisher, the editors and the reviewers. Any product that may be evaluated in this article, or claim that may be made by its manufacturer, is not guaranteed or endorsed by the publisher.

## Footnotes

^1^C40 Cities Climate Leadership Group (C40) is a network of the world’s megacities engaging in reducing greenhouse gas emissions (13).
